# Profound seasonal shrinking and regrowth of the ossified braincase in phylogenetically distant mammals with similar life histories

**DOI:** 10.1038/srep42443

**Published:** 2017-02-13

**Authors:** Dina K. N. Dechmann, Scott LaPoint, Christian Dullin, Moritz Hertel, Jan R. E. Taylor, Karol Zub, Martin Wikelski

**Affiliations:** 1 Max Planck Institute for Ornithology, Department of Migration and Immuno-ecology, 78315 Radolfzell, Germany; 2 University of Konstanz, Department of Biology, 78457 Konstanz, Germany; 3 Lamont-Doherty Earth Observatory, Columbia University, Department of Earth and Environmental Sciences, Palisades, New York 10964, USA; 4 University Hospital Göttingen, Institute for Diagnostic and Interventional Radiology, 37075 Göttingen, Germany; 5 Max Planck Institute for Ornithology, Department of Behavioural Neurobiology, 82319 Seewiesen, Germany; 6 University of Białystok, Institute of Biology, 15-245 Białystok, Poland; 7 Mammal Research Institute, 17-230 Białowieża, Poland

## Abstract

Ontogenetic changes in skull shape and size are ubiquitous in altricial vertebrates, but typically unidirectional and minimal in full-grown animals. Red-toothed shrews exhibit a rare exception, where the shape, mass and size of the skull, brain, and several major organs, show significant bidirectional seasonal changes. We now show a similar but male-biased shrinking (16%) and regrowth (8%) in the standardized braincase depth of least weasels (*Mustela nivalis*). Juvenile weasels also exhibit a growth overshoot, followed by a shrinkage period lasting until the end of their first winter. Only male weasels then regrow during their second summer. High-resolution CT scans suggest areas of the skull are affected differently during shrinking and regrowth in both species. This suggests multiple evolutionary drivers: while the shrinking likely facilitates survival during seasonal low resource availability in these high-metabolic mammals with year-round activity, the regrowth may be most strongly influenced by high investment into reproduction and territories, which is male-biased in the weasels. Our data provide evidence for convergent evolution of skull and thus brain shrinkage and regrowth, with important implications for understanding adaptations to changing environments and for applied research on the correlated changes in bone structure, brain size and the many other affected organs.

Population-level body size (i.e., skeletal or skull dimensions) can vary as a consequence of biased selection, for example in voles (*Microtus oeconomus*) and least weasels (*Mustela nivalis*) where harsh winter conditions favour the survival of relatively small individuals^1^,^2^. At the individual level, growth of body size typically slows down drastically or stops once individuals reach adulthood, aside from some bone degeneration in very old individuals^3^,^4^. Examples where body size does continue to change are usually in response to harsh conditions and not expressed in a genetically fixed pattern. For example some amphibians and tortoises reduce body length under drought conditions^5^,^6^. A notable exception to such unidirectional shrinking is the Galapagos marine iguana (*Amblyrhynchus cristatus*) where individuals exhibit reversible body length changes in response to episodically limited resource availability^7^. In contrast, several species of red-toothed shrews reach a first maximum size as juveniles, then drastically decrease and later even regrow the size of their body, various organs, and in particular the brain and skull in a fixed seasonal pattern. For example in the common shrew (*Sorex araneus*), skull size, brain mass and several other organs change by up to 20% between seasons (Figs 1b and 2 8,9,10). Although many species exhibit juvenile skull shape and size changes, an absolute growth overshoot was only known for juvenile shrews and mustelids^11^,^12^. Some evidence exists for an ensuing decrease in both braincase depth and brain mass in domesticated mustelids^11^,^13^ and the braincase in free-ranging least weasels^14^, but the later regrowth has to date only been studied in red-toothed shrews.

However, if these changes are adaptive and driven by seasonal changes, as the tight link of the size extremes to winter (smallest size) and spring (regrowth) indicates, they should manifest in more than one taxon. Thus, we quantified seasonal changes in skull length and braincase depth, a common measure for detecting seasonal size change in shrews, in the least weasel, one of the mustelid species that shows a juvenile skull size overshoot similar to juvenile shrews. Weasels (family Mustelidae, order Carnivora) and red-toothed shrews (family Soricidae, order Eulipotyphla) diverged during the paleocene^15^, yet share several life history traits, including: (i) small size, (ii) limited ability to compensate for heat loss^16^,^17^, (iii) an extremely high metabolism that is always near the maximum^17^,^18^, resulting in constant requirements for high quality food^19^, (iv) a short lifespan^19^, and (v) year-round activity^17^,^20^ without the ability to optimize energy use through torpor. Detecting reversible size patterns in a greater diversity of species will facilitate rigorous hypothesis testing, improving our understanding of the mechanisms, consequences, and evolution of this intriguing phenomenon. This would not only be especially interesting in the context of whether fundamental evolutionary patterns are deterministic or phylogenetically contingent^21^,^22^, but also have profound implications for medical research on the underlying mechanisms in the ossified bone, as well as all other affected organs and tissues.

## Results and Discussion

**Figure 1:**
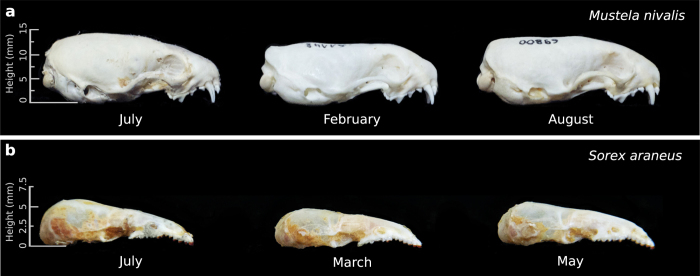
Skulls of male *M. nivalis* (**a**) and *S. araneus* (**b**) showing the absolute juvenile (July) growth overshoot, the winter minima (February and March), and the regrowth (August and May) in braincase depth. See methods.

**Figure 2:**
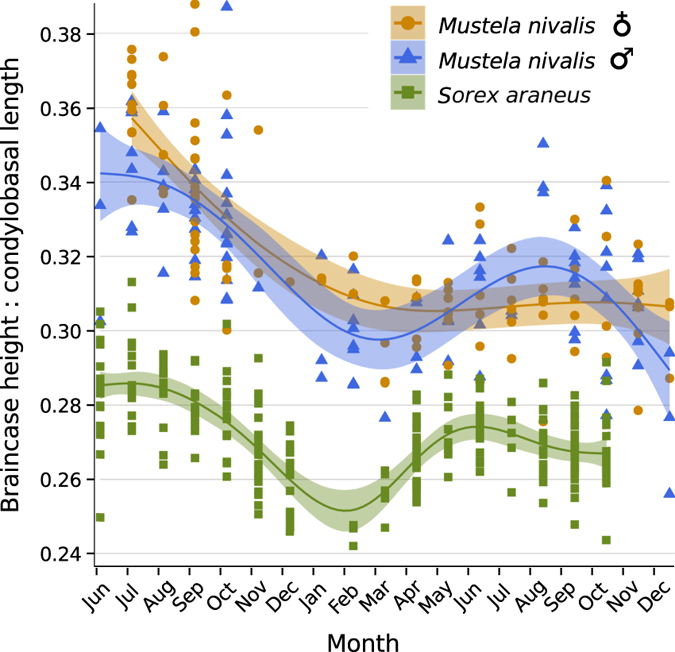
Standardized braincase depths for *M. nivalis* and *S. araneus* plotted along a lifetime axis. Green squares, orange circles, and blue triangles indicate standardized braincase depths of *S. araneus*, and female and male *M. nivalis*, respectively. Solid lines represent generalized additive model predictions for standardized braincase depth with shaded, Bayesian confidence intervals.

Our data provide the first evidence for a full, but male-biased cycle of seasonal reversible size change in a carnivore (Figs 1a and 2). We identified an age and seasonal pattern in the standardized braincase depth of extracted skulls of male (n = 130), but not female (n = 120), weasels (Fig. 2). As in shrews, both sexes exhibit a juvenile growth overshoot, and then decrease by 15.5% until the following spring. Following spring, male weasels exhibit a remarkable regrowth in their second summer, achieving mean standardized braincase depths 8.3% greater than those during the preceding winter, matching the results for shrews (n = 244; 9.9% decrease followed by a 5.4% regrowth to the second peak; Fig. 2).

Our generalized additive model also suggested different braincase depth patterns between female and male shrews, with females exhibiting a more sinuous seasonal pattern than males. However, the sample size for male specimens collected between September and March; i.e., during the predicted winter minimum, was small (Dataset S1). Thus we present shrew results without distinguishing between the sexes (Fig. 2).

The minimal overlap in the data between seasons offers strong support that these patterns are not the result of population-level selection events (Fig. 2). Further, adults of both species exhibit a second depth decrease near the end of the second summer after the end of their primary reproductive period. Similarly, the spring regrowth also starts before reproduction^23^ and thus cannot be the result of new individuals being added to the population.

**Figure 3:**
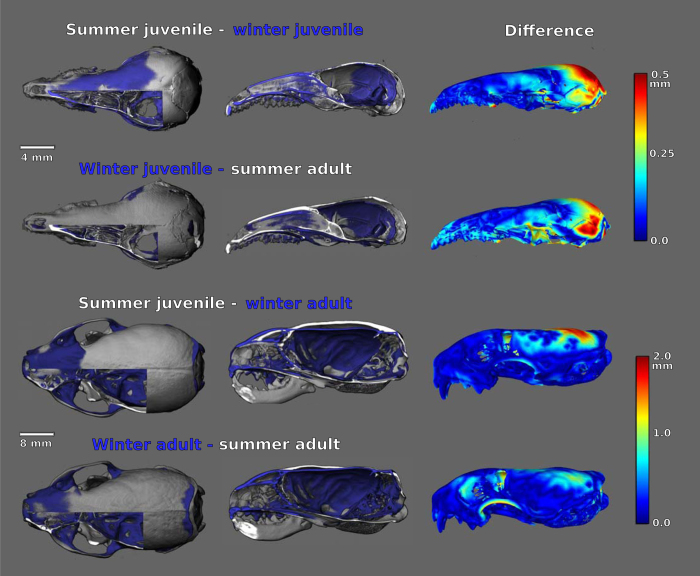
CT overlays (left and center) and heat maps (right) of seasonal changes in the skulls of shrews (top) and weasels (bottom). All skulls were aligned by the upper toothrow. Skulls used are the same as in Fig. 1. Overlays: dorsal and lateral view of the decrease (summer juvenile (white) to winter subadult (blue)) and increase (winter subadult (blue) to regrown summer adult (white)). Heat maps: lateral view only; increasing magnitude of change from blue to red.

Overlays and heat maps of high resolution CT scans (Fig. 3) allow direct 3D visualization of the change between seasons. They suggest that the size changes in both species also involve shape changes. This difference appears even more striking in the shrews but is clearly visible also in the weasels. Very obvious in the weasels is the initially thick skull, which becomes much thinner in winter and never completely regains its initial thickness. These changes, if anything, should lessen the corresponding changes in brain size, as the skull is thinnest when the braincase height is the smallest. This is also reflected in the much more structured bone surface with the development of stabilization lines and muscle attachment sites. In the shrews the most striking process is the dramatic lift of the base of the skull from first summer to winter and second summer. Although our simple linear measurements identified a depth change, how this change is occurring is much more evident from these visualizations. Much of the important change occurs near the lambdoidal suture, which moves upward while changing shape in the decrease from first summer to winter. As there is no easy methodological way to remove this “bending” effect from the surface models, the distance maps need to be seen as a representation of the total change in shape by shrinkage and bending and not as a direct measure of the shrinkage effect. In conclusion, it appears that the environmental conditions and energetic constraints that most likely drive this phenomenon may affect the skull and consequently the brain and its regions in different ways. However the changes are obviously complicated and will require much more detailed and powerful research approaches to be fully quantified and understood.

Both shrews and weasels have much higher metabolisms than similarly sized mammals^17^,^18^, thus any absolute individual size reductions, particularly in energetically expensive organs such as the brain, should reduce absolute energy requirements. This is true between individuals: smaller individuals of both shrews and weasels use less absolute energy than larger individuals^2^,^24^. The size change is likely an adaptation to fluctuations in the availability and quality of resources (*sensu*^25^). Both species use pulsing or cyclical resources and must find new sources of food in spring^23^,^26^. The shrinkage and re-growth in skull and presumably brain size may be a result of conflicting selection pressures of low resource availability and requirements of a larger behavioural repertoire during certain life stages^27^ and the discovery of this cycle in the phylogenetically distant weasels adds credibility especially to the latter. The large skull during the first summer coincides with juvenile dispersal, high activity and probably the establishment of winter territories and shelters. The anticipatory autumn shrinking would prepare animals for the arrival of harsh winter conditions, when both species exhibit less locomotor activity, at least partly due to thermoregulatory constraints^28^,^29^. An anticipatory spring increase may be impossible – instead individuals increase along with improving resources to prepare for the reproductive season and its correlated behaviours near the end of their short lives^17^,^30^. However, while female and male shrews defend territories equally as violently^23^, female weasels may invest more into reproduction and caring for young and may not be able to allocate energetic resources to a larger brain in the spring. Similar to other seasonally driven convergent morphological changes, such as changing fur colour^31^, the autumn decrease is induced before the actual selective pressure is activated (Fig. 2). Thus we hypothesize that this seasonal size pattern (i.e., Dehnel’s Phenomenon^8^) is largely genetically fixed even if modulated by the severity of environmental conditions. In fact, captive shrews and mustelids with consistent food availability, quality, and ambient temperatures continue to exhibit a weaker form of the skull flattening, but not the later regrowth^13^,^32^.

Postnatal changes in skull *shape* are common in highly altricial species, but they are usually ontogenetic^3^. Changes in brain size, in response to the environment or experimental treatment, are usually less than 5%^33^. Adult brain size can continue to vary and in the case of some songbirds at similar or even greater scales of magnitude^34^. In contrast to birds, where similar reversible changes in brain size (but not other structures that show associated changes in the shrew) have been demonstrated, there is no evidence for the establishment of new neurons in shrews^35^ and the changes cannot be explained by a change in water or fat content alone^36^. The magnitude of relative braincase decrease and regrowth that we report here was previously only documented in *S. araneus* and now for the first time in *M. nivalis*. While alternative or additional drivers cannot be excluded based on current evidence, an altricial life style and an energetically limited physiology may be preconditions for the evolution of these seasonal reversible size changes, and may explain why this phenomenon is apparently rare. In addition, the change in shrew and mustelid (i.e., ranch mink; *M. vison*) brain size is accompanied by a complete reorganization of the various brain regions^10^,^13^. For example, Yaskin^37^ reports a 20% volume change of the hippocampus with strong assumed consequences on the animals’ cognitive abilities. More research is warranted to better understand why the bidirectional change in brain size includes re-organization during all stages.

Our data suggest a link between high-energetic life histories and seasonally variable resource availabilities and qualities, improving our understanding of how small high-energetic animals deal with massively changing, but largely predictable environments. Future research efforts should include repeated measures of individuals to exclude potential selection effects. However our data and results are robust for the following reasons: (i) we controlled for sex (statistically) and geographic sampling effects, (ii), the greatest changes occur during a time when there is little (weasels) to no (shrews) cohort overlap; and (iii) the CT scans depict how and where these changes occur, increasing the plausibility of the intra-individual change. We also need more inter-specific comparisons to better document the broader phylogenetic scope of this phenomenon that our data imply. Efforts to quantify intra-specific and -individual as well as sex-specific variation in the magnitude of these seasonal changes and their correlation with potential drivers such as climate, photoperiod, or local food availability will facilitate predictions on the impacts of global change patterns. Finally, a better comparative understanding of the documented changes in hormone physiology and, the digestive system (both reviewed in^38^), ossified bone^39^, and the regrowth and reorganization of the brain regions^10^,^13^ will be of great interest to medical research.

## Methods

### Data

The shrew specimens first used to describe the reversible size patterns (i.e. Dehnel's Phenomenon) were collected from Białowieża National Park in Poland^8^. We re-measured these and additional specimens (collected between 23 September 1946 and 11 December 1947) in the collection at the Mammal Research Institute, Białowieża (Dataset S1).

Shrews had been aged by the collectors and we verified this by checking tooth wear and coloration (less worn teeth with larger red tips in juveniles than in adults^40^ as well as the colour of the fur, as skins were also present for all specimens (juveniles more grey and less distinctly tricolour, also overall smaller pelts^8^). Because shrews live approximately one year, adults were only found from late March to late October.

To account for biogeographic patterns in weasel body size^26^ and local climate, we only included weasel specimens collected in or near Białowieża, Poland (i.e., latitudes > 52° and longitudes > 21° within Poland). We classified weasel specimens into young adult and old adult, observing morphological guidelines for weasels to match the original literature for shrews^8^,^14^,^26^. Young adult weasel skulls were generally not fully ossified nor did they have completely fused sutures, with evidence of nuchal or sagittal crests developing, and with a generally smooth and rounded braincase posterior. Old adult skulls were fully ossified with fully fused sutures, developed nuchal crests and often showing signs of damage to the postorbital area by parasitic worms (*Skrjabingylus nasicola)*. Using these aging criteria produced young adult weasels that had collection dates between mid-June and mid-January and old adults throughout the year.

We used the same six specimens from the collection at the Mammal Research Institute for all visualizations (Figure 1 and 3): catalogue numbers are as follows, weasels: July overshoot juvenile: 38738, decreased February subadult: 51148, regrown August adult: 69800, and shrews: July overshoot juvenile 1240, decreased March subadult 507, regrown May adult 2730.

We recorded braincase depth (perpendicularly from the basioccipital to the top of the braincase, omitting the auditory bullae and sagittal crest when present) with digital calipers (+/−0.01 mm). We standardized this with condylobasal length (measured from the posterior of the occipital condyles to the anterior tip of the premaxilla) to make direct comparisons between species and sexes and also to reduce potential between-year size variation enhanced by the 51-year span during which the weasel specimens were collected. Condylobasal length, a commonly used standardization measure, showed no age or seasonal patterns (Figure S1). A single observer (SDL) took all measurements blind to the date of collection to reduce potential bias.

Seasonal changes in mass of extracted brains have been previously reported in the shrews^36^. Comparable data are not available for weasels. Dried tissue residue inside the braincase prevented us from measuring weasel braincase volume.

### Statistical analyses

We used generalized additive models (GAM^41^,^42^ to test whether standardized braincase depths of shrews and weasels demonstrate age and seasonal patterns. GAMs are semi-parametric generalizations of linear regression models that allow for both linear and non-linear relationships between the response and predictors and include a smoothing function that penalizes the model likelihood for the addition of each smooth term and the amount of ‘wiggliness’ in each model prediction^41^. Because we had an *a priori* expectation for the presence and shape of a seasonal braincase depth pattern, we used the GAM to model this pattern and test whether weasel and shrew braincase depths exhibited similar seasonal patterns. To do this, we added a cubic regression spline smoother as a penalty term on an age-adjusted date of collection and set the number of knots to five; forcing the model to attempt to identify a seasonal pattern. The age-shifted date of collection was created by adding 365 to the Julian day of the year of the date of collection to old adult specimens only, and then subtracting one less than the lowest Julian day of the year of collection of young adults from all specimens. This produced maximum values of 511 and 544 for shrews and weasels, respectively, with a minimum of 1 for both species. We ran these models for each species separately and included sex as a factor.

To determine whether potential seasonal patterns differed in shape between the sexes of each species, we ran separate models with a smooth term per sex^43^ and performed an ANOVA on the model with the smooth term applied to each sex separately and without. Instances where applying the smooth term to each sex separately improved the model performance would suggest that the response is better described for each sex separately, rather than combined; i.e., in addition to sex differences in the estimate, but also potential differences in the shape of model fit. Lastly, we inspected the residual diagnostics of the optimal models to assess the normality and homogeneity of the residuals and the model fit. All analyses were conducted within program R version 3.1.3^44^ using the *mgcv* package version 1.8–6^43^.

To visualize these seasonal changes, we CT-scanned the skull specimens shown in Figures 1 and 2 and created overlays (Fig. 3). Micro CT systems are optimized for a specific range of sample sizes, and thus we used two different systems. The bigger weasel skulls were scanned utilizing the QuantumFX low dose *in-vivo* micro CT (Perkin Elmer) operated with the following parameters: tube voltage = 90 kVp, tube current = 200 μA, field-of-view = 40 × 40 mm^2^, resulting in 3D datasets with a reconstructed voxel size of 80 × 80 × 80 μm^3^. The much smaller shrew skulls with their thin bones required a higher spatial resolution, i.e. the eXplore Locus SP specimen micro CT (GE HealthCare). The shrew data sets were reconstructed with a voxel size of 16 × 16 × 16 μm^3^.

We then processed the generated CT datasets with the 3D visualization and analysis software Scry v6.0 (Kuchel & Sautter GbR). We chose the arithmetic mean between the average value of bone and the background as threshold for calculation of the skull surface. Polygonization was performed utilizing a marching cubes algorithm^45^ implemented within Scry v 6.0 (Kuchel & Sautter GbR) resulting in triangle surface meshes archived in stereo-lithography (STL) file format. We aligned the upper toothrow of skulls of two consecutive stages of the size change (i.e. large summer juvenile (white) with size-decreased winter subadult (blue), and winter subadult (blue) with regrown summer adult (white); Fig. 3) to visualize the changes within the skulls of each species.

### Ethics

No live animals were used during this research.

### Data availability

The dataset supporting this article has been uploaded as part of the Supplementary Material.

## Additional Information

**How to cite this article**: Dechmann, D. K. N. *et al*. Profound seasonal shrinking and regrowth of the ossified braincase in phylogenetically distant mammals with similar life histories. *Sci. Rep.* **7**, 42443; doi: 10.1038/srep42443 (2017).

**Publisher's note:** Springer Nature remains neutral with regard to jurisdictional claims in published maps and institutional affiliations.

## Supplementary Material

Supplementary Information

Supplementary Dataset

## References

[b1] Zub K., Borowski Z., Szafranska P. A., Wieczorek M. & Konarzewski M. Lower body mass and higher metabolic rate enhance winter survival in root voles, Microtus oeconomus. Biol. J. Linnean Soc. 113, 297–309, doi: 10.1111/Bij.12306 (2014).

[b2] Zub K., Szafranska P. A., Konarzewski M. & Speakman J. R. Effect of energetic constraints on distribution and winter survival of weasel males. J. Anim. Ecol. 80, 259–269, doi: 10.1111/j.1365-2656.2010.01762.x (2011). 21039480

[b3] Gould S. J. Allometry and size in ontogeny and phylogeny. Biol. Rev. 41, 587-+, doi: 10.1111/j.1469-185X.1966.tb01624.x (1966). 5342162

[b4] Emerson S. B. & Bramble D. M. In The skull Vol. 3 (eds J. Hanken & B. K. Hall) 384ff (Chicago University Press, 1993).

[b5] Bendik N. F. & Gluesenkamp A. G. Body length shrinkage in an endangered amphibian is associated with drought. J. Zool. 290, 35–41, doi: 10.1111/Jzo.12009 (2013).

[b6] Loehr V. J. T., Hofmeyr M. D. & Henen B. T. Growing and shrinking in the smallest tortoise, *Homopus signatus signatus*: the importance of rain. Oecologia 153, 479–488, doi: 10.1007/S00442-007-0738-7 (2007). 17453250

[b7] Wikelski M. & Thom C. Marine iguanas shrink to survive El Nino - Changes in bone metabolism enable these adult lizards to reversibly alter their length. Nature 403, 37–38, doi: 10.1038/47396 (2000). 10638740

[b8] Dehnel A. Studies on the genus *Sorex* L. Ann. Univ. Mariae Curie-Skodowska C 4, 17–102 (1949).

[b9] Pucek Z. Seasonal changes in the braincase of some representatives of the genus *Sorex* from the Palaearctic. J. Mammal. 44, 523–536 (1963).

[b10] Yaskin V. A. Variation in brain morphology of the common shrew. Carnegie Mus. Nat. Hist. Special Pub. 5, 155–161 (1994).

[b11] Apfelbach R. & Kruska D. Postnatal development of the brain of the ferret *Mustela putorius* F Furo (Mustelidae, Mammalia). Int. J. Mammal. Biol. 44, 127–131 (1979).

[b12] Kruska D. Über die postnatale Hirnentwicklung beim Farmnerz *Mustela vison* f. dom. Mustelidae: Mammalia) Z. Säugetierkunde 42, 240–255 (1977).

[b13] Kruska D. Evidence of decrease in brain size in ranch mink, *Mustela vison* F DOM, during subadult postnatal ontogeny. Brain Behav. Evol. 41, 303–315 (1993). 8324619 10.1159/000113851

[b14] Schmidt K. Skull variability of *Mustela nivalis* Linnaeus, 1766 in Poland. Acta Theriol. 37, 141–162 (1992).

[b15] O’Leary M. A. . The placental mammal ancestor and the post-K-Pg radiation of placentals”. Science 339, doi: 10.1126/science.1229237 (2013). 23393258

[b16] Taylor J. R. E., Rychlik L. & Churchfield S. Winter reduction in body mass in a very small, nonhibernating mammal: consequences for heat loss and metabolic rates. Physiol. Biochem. Zool. 86, 9–18, doi: 10.1086/668484 (2013). 23303317

[b17] Zub K., Szafranska P. A., Konarzewski M., Redman P. & Speakman J. R. Trade-offs between activity and thermoregulation in a small carnivore, the least weasel *Mustela nivalis*. Proc. R. Soc. B 276, 1921–1927, doi: 10.1098/Rspb.2008.1936 (2009). PMC2674502 19324766

[b18] Ochocinska D. & Taylor J. R. E. Living at the physiological limits: Field and maximum metabolic rates of the common shrew (*Sorex araneus*). Physiol. Biochem. Zool. 78, 808–818, doi: 10.1086/431190 (2005). 16096983

[b19] Gliwicz J. & Taylor J. R. E. Comparing life histories of shrews and rodents. Acta Theriol. 47, 185–208 (2002).

[b20] Taylor J. R. E. In Evolution of shrews (eds J. M. Wojcik & M. Wolsan) 309–346 (Polish Academy of Sciences, 1998).

[b21] Mahler D. L., Ingram T., Revell L. J. & Losos J. B. Exceptional convergence on the macroevolutionary landscape in island lizard radiations. Science 341, 292–295 (2013). 23869019 10.1126/science.1232392

[b22] Gillespie R. G. Adaptive radiation: convergence and non-equilibrium. Current Biology 23, R71–R74 (2013). 23347943 10.1016/j.cub.2012.11.052

[b23] Churchfield S. The natural history of shrews. (Christopher Helm Ltd., 1990).

[b24] Szafranska P. A., Zub K. & Konarzewski M. Seasonal variation of resting metabolic rate and body mass in free-living weasels *Mustela nivalis*. Physiol. Biochem. Zool. 86, 791–798, doi: 10.1086/673286 (2013). 24241075

[b25] McNab B. K. Geographic and temporal correlations of mammalian size reconsidered: a resource rule. Oecologia 164, 13–23, doi: 10.1007/s00442-010-1621-5 (2010). 20364270

[b26] King C. M. & Powell R. A. The Natural History of Weasels and Stoats; Ecology, Behavior, and Management. 2 edn, 446 (Oxford University Press, 2007).

[b27] Piersma T. & Drent J. Phenotypic flexibility and the evolution of organismal design. Trends Ecol. Evol. 18, 228–233, doi: 10.1016/S0169-5347(03)00036-3 (2003).

[b28] Rychlik L. In Evolution of shrews (eds J. M. Wojcik & M. Wolsan) 347–406 (Mammal Research Institute, 1998).

[b29] Zub K., Sonnichsen L. & Szafranska P. A. Habitat requirements of weasels *Mustela nivalis* constrain their impact on prey populations in complex ecosystems of the temperate zone. Oecologia 157, 571–582, doi: 10.1007/S00442-008-1109-8 (2008). 18629542

[b30] Stockley P., Searle J. B., Macdonald D. W. & Jones C. S. Alternative reproductive tactics in male common shrews: relationships between mate-searching behaviour, sperm production, and reproductive success as revealed by DNA fingerprinting. Behav. Ecol. Sociobiol. 34, 71–78 (1993).

[b31] Mills L. S. . Camouflage mismatch in seasonal coat color due to decreased snow duration. Proc. Natl. Acad. Sci. USA 110, 7360–7365, doi: 10.1073/Pnas.1222724110 (2013). 23589881 PMC3645584

[b32] Pucek Z. Morphological changes in shrews kept in captivity. Acta Theriol. 8 (1964).

[b33] Bedi K. S. & Bhide P. G. Effects of environmental diversity on brain morphology. Early Hum Dev 17, 107–143, doi: 10.1016/S0378-3782(88)80001-X (1988). 3061774

[b34] Nottebohm F. Neuronal replacement in adulthood. Ann. New York Acad. Sci. 457, 143–161 (1985). 3913361 10.1111/j.1749-6632.1985.tb20803.x

[b35] Bartkowska K., Djavadian R. L., Taylor J. R. E. & Turlejski K. Generation recruitment and death of brain cells throughout the life cycle of *Sorex* shrews (Lipotyphla). Europ. J. Neurosci. 27, 1710–1721, doi: 10.1111/j.1460-9568.2008.06133.x (2008). 18380668

[b36] Pucek M. Water contents and seasonal changes of the brain-weight in shrews. Acta Theriol. **X**, 353–367 (1965).

[b37] Yaskin V. A. In Advances in the biology of shrews. II. [Special Publication of the International Society of Shrew Biologists Number 01.] (eds J. F. Merritt, S. Churchfield, R. Kock, D. Hutterer & B. I. Sheftel) 373–385 (International Society of Shrew Biologists, 2005).

[b38] Hyvärinen H. In Wintering ecology of small mammals. (ed. Merritt J. F.) 139–148 (Special Publication of the Carnegie Museum of Natural History 10, 1984).

[b39] Pucek Z. Untersuchungen über die Veränderlichkeit des Schädels im Lebenszyklus von *Sorex araneus araneus* L. Ann. Univ. Mariae Curie-Skodowska C 9, 163–211 (1955).

[b40] Pankakoski E. Variation in the tooth wear of the shrews *Sorex araneus* and *S. minutus*. in Ann. Zool. Fennici. 26 445–457 (1989).

[b41] Hastie T. & Tibshirani R. Generalized additive models. Statistical science, 297–310 (1986). 10.1177/096228029500400302 8548102

[b42] Wood S. N. Generalized Additive Models: an introduction with R. (Chapman and Hall, 2006).

[b43] mgcv: mixed GAM computation vehicle with GCV/AIC/REML smoothness estimation v. 1.8-6 (2015).

[b44] R. a language and environment for statistical computing (R Foundation for Statistical Computing, Vienna, 2015).

[b45] Lorensen W. E. & Cline H. E. Marching cubes: a high resolution 3D surface construction algorithm. ACM siggraph computer graphics 21 (1987).

