# Supplementary material for: Profound seasonal shrinking and regrowth of the ossified braincase in phylogenetically distant mammals with similar life histories
Source: Sci Rep. 2017 Feb 13;7:42443. doi: 10.1038/srep42443 (PMC5304206; doi:10.1038/srep42443)
Supplement: Supplementary Information [file srep42443-s1.pdf]

Profound seasonal shrinking and regrowth of the ossified braincase in phylogenetically distant mammals with similar life histories

Dina K. N. Dechmann[1,2*†], Scott LaPoint[1,3†], Christian Dullin[4], Moritz Hertel[5], Jan R. E. Taylor[6], Karol Zub[7], Martin Wikelski[1,2]

Supplementary Materials:

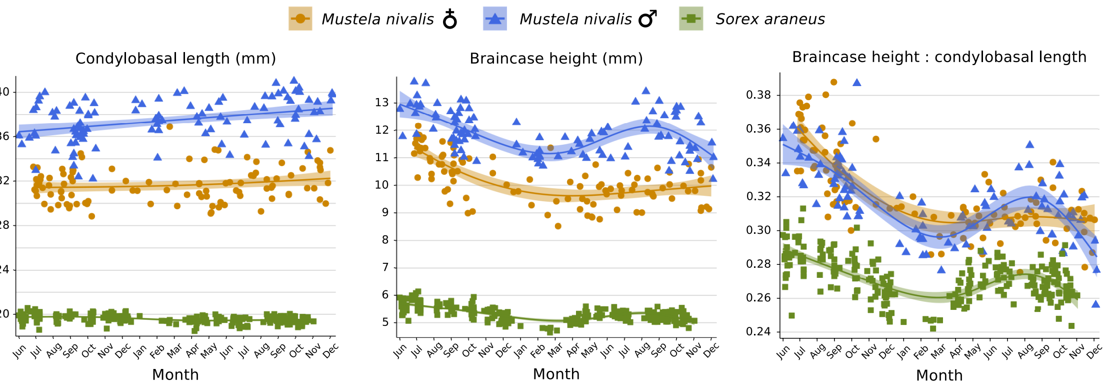

Figure S1. Generalized additive model predictions for seasonal patterns in the condylobasal lengths (left), braincase depths (center), and standardized braincase depths (right) for female weasels (orange and circles), male weasels (blue and triangles), and shrews (green and squares). Here we show that the seasonal pattern in standardized braincase depth is not driven by disproportionate nor independent changes in condylobasal length or braincase depth alone.
